# Clonal Spread and Intra- and Inter-Species Plasmid Dissemination Associated With *Klebsiella pneumoniae* Carbapenemase-Producing Enterobacterales During a Hospital Outbreak in Barcelona, Spain

**DOI:** 10.3389/fmicb.2021.781127

**Published:** 2021-11-18

**Authors:** Marta Marí-Almirall, Núria Ferrando, Mariana José Fernández, Clara Cosgaya, Joaquim Viñes, Elisa Rubio, Anna Cuscó, Laura Muñoz, Martina Pellice, Andrea Vergara, Irene Campo, Laura Rodríguez-Serna, Gemina Santana, Ana Del Río, Olga Francino, Pilar Ciruela, Frederic Ballester, Francesc Marco, José Antonio Martínez, Álex Soriano, Cristina Pitart, Jordi Vila, Ignasi Roca, MERCyCAT Study Group, Pepa Pérez Jove

**Affiliations:** Catlab, Centre Analítiques Terrassa AIE; Consorci del Laboratori Intercomarcal de l’Alt Pendès, l’Anoia i el Garraf; Corporació de Salut del Maresme i la Selva; Hospital de Mataró; Hospital General de Granollers; Hospital General de Vic; Hospital General del Parc Sanitari Sant Joan de Déu; Hospital Sant Joan de Déu de Barcelona; Hospital Sant Joan de Déu de Manresa; Hospital Sant *Pau*I Santa Tecla; Hospital Universitari Joan XXIII de Tarragona; Hospital Universitari Sant Joan de Reus; ^1^Laboratory of Antimicrobial Resistance, ISGlobal, Hospital Clínic–Universitat de Barcelona, Barcelona, Spain; ^2^Department of Clinical Microbiology, Hospital Clínic–Universitat de Barcelona, Barcelona, Spain; ^3^Molecular Genetics Veterinary Service, Universitat Autònoma de Barcelona, Barcelona, Spain; ^4^Vetgenomics, PRUAB, Universitat Autònoma de Barcelona, Barcelona, Spain; ^5^Department of Infectious Diseases, Hospital Clínic–Institut d’Investigacions Biomèdiques August Pi i Sunyer, Universitat de Barcelona, Barcelona, Spain; ^6^Department of Preventive Medicine and Epidemiology, Hospital Clínic–Universitat de Barcelona, Barcelona, Spain; ^7^Public Health Agency of Catalonia (ASPCAT), Generalitat de Catalunya, Barcelona, Spain; ^8^CIBER de Epidemiología y Salud Pública, Instituto de Salud Carlos III, Madrid, Spain; ^9^Hospital Universitari Sant Joan de Reus-Laboratori de Referència del Camp de Tarragona i de les Terres de l’Ebre, Reus, Spain

**Keywords:** *Klebsiella*, outbreak, antibiotic resistance, carbapenemase, epidemiology, plasmid, high-risk clone, KPC

## Abstract

**Objectives:** The study aimed to characterize the clonal spread of resistant bacteria and dissemination of resistance plasmids among carbapenem-resistant Enterobacterales at a tertiary hospital in Catalonia, Spain.

**Methods:** Isolates were recovered from surveillance rectal swabs and diagnostic samples. Species identification was by matrix-assisted laser desorption ionization-time time of flight mass spectrometry (MALDI-TOF MS). Molecular typing was performed by pulsed-field gel electrophoresis (PFGE) and multi-locus sequence typing (MLST). Antimicrobial susceptibility was assessed by gradient-diffusion and carriage of *bla* genes was detected by PCR. Plasmid typing, conjugation assays, S1-PFGE studies and long-read sequencing were used to characterize resistance plasmids.

**Results:** From July 2018 to February 2019, 125 *Klebsiella pneumoniae* carbapenemase (KPC)-producing Enterobacterales were recovered from 101 inpatients from surveillance (74.4%) or clinical samples (25.6%), in a tertiary hospital in Barcelona. Clonality studies identified a major clone of *Klebsiella pneumoniae* belonging to sequence type ST15 and additional isolates of *K. pneumoniae*, *Escherichia coli* and *Enterobacter* sp. from different STs. All isolates but one carried the *bla*_KPC–2_ allelic variant. The *bla*_KPC–2_ gene was located in an IncFIIk plasmid of circa 106 Kb in a non-classical Tn*4401* element designated NTE_KPC_-pMC-2-1. Whole-genome sequencing revealed different rearrangements of the 106 Kb plasmid while the NTE_KPC_-pMC-2-1 module was highly conserved.

**Conclusion:** We report a hospital outbreak caused by the clonal dissemination of KPC-producing ST15 *K. pneumoniae* but also the intra- and inter-species transmission of the *bla*_KPC–2_ gene associated with plasmid conjugation and/or transposon dissemination. To our knowledge, this is the first report of an outbreak caused by KPC-producing Enterobacterales isolated from human patients in Catalonia and highlights the relevance of surveillance studies in the early detection and control of antibiotic resistant high-risk clones.

## Introduction

The rapid emergence of bacterial pathogens presenting resistance to multiple antimicrobial agents (MDR) together with the decreasing trend in the development of new antimicrobial compounds constitute an extremely serious threat to public health (Roca et al., 2015). Resistance rates are particularly alarming among Gram-negative bacteria (GNB) as available treatment options are severely impaired. In 2016 a report commissioned by the UK Prime minister, known as the “O’Neill report,” attributed 700.000 annual human deaths worldwide to microbial infections caused by antimicrobial resistant (AMR) pathogens (O’neill, 2016). The same report estimated that this figure would rise to 10 million annual deaths by 2050 unless there is a global effort to tackle AMR. Likewise, organizations such as the US Centers for Disease Control and Prevention (CDC), the European Centre for Disease Prevention and Control (ECDC) and the World Health Organization (WHO) are considering infections caused by MDR bacteria as an emergent global disease and a major public health problem (Roca et al., 2015). According to their mortality, health-care and community burden, prevalence of resistance and treatability, carbapenem-resistant Enterobacterales have been included into the 2017 WHO priority list of antibiotic-resistant bacterial pathogens and currently constitute one of the major public health threats worldwide (Tacconelli et al., 2017).

Among carbapenem-resistant Enterobacterales, *Klebsiella pneumoniae* stands out as a formidable nosocomial pathogen causing several infections associated with high mortality rates (Tumbarello et al., 2012). In Europe, carbapenem-resistant *K. pneumoniae* accounted for almost 16,000 infections and more than 2,000 deaths in 2015 (Cassini et al., 2018). Carbapenem-resistance is usually associated with the carriage of genes encoding carbapenem-hydrolyzing enzymes, most of which are commonly located within conjugative plasmids and, therefore, are easily disseminated among nosocomial pathogens (Bonomo et al., 2017). Predominant carbapenemases in Europe include OXA-48 oxacillinase, New Delhi metallo-β-lactamase (NDM) and *Klebsiella pneumoniae* carbapenemase (KPC), among others. In Spain, OXA-48 is currently the predominant mechanism associated with the dissemination of carbapenem-resistant Enterobacterales (Grundmann et al., 2017). NDM-producing bacteria are reported less frequently in Spain but their presence is increasing and associated with a few clonal lineages (Marí-Almirall et al., 2021) while KPC-producing Enterobacterales are also sporadic in Spain, and only a few outbreaks have been reported in Madrid and Andalusia (Oteo et al., 2016).

*Klebsiella pneumoniae* carbapenemase is likely one of the most common mechanisms of resistance to carbapenems in *K. pneumoniae*, and the worldwide spread of the *bla*_KPC_ gene was initially associated with the dissemination of a few *K. pneumoniae* clonal lineages within the ST258 clonal group (CG258), a particular plasmid backbone (pKpQIL) and a highly conserved Tn*4401* transposon (Chen et al., 2014). More recently *bla*_KPC_ has been found on a variety of clonal lineages and plasmids from different incompatibility groups (Wyres et al., 2020), and although it is usually associated with a composite Tn*4401* transposon, some structural variations have also been described (Shen et al., 2009; Naas et al., 2012; Chmelnitsky et al., 2014). In this retrospective study we have examined the rapid dissemination of KPC-producing Enterobacterales within a tertiary hospital in Barcelona and we have investigated the inter- and intra-species spread of carbapenem resistance.

## Materials and Methods

This study included 125 carbapenem-resistant isolates of Enterobacterales collected from July 2018 to February 2019 at one tertiary hospital in Barcelona, Spain. Strains were recovered from surveillance and clinical samples (Supplementary Table 2). Identification at species level was performed by matrix-assisted laser desorption ionization-time time of flight mass spectrometry (MALDI-TOF MS) in a Microflex LT benchtop instrument (Bruker Daltonics) operated in linear positive mode.

### Antimicrobial Susceptibility Testing and Detection of Resistance

Antimicrobial susceptibility was assessed by gradient diffusion (*E*-test, BioMérieux, Spain) on Muller-Hinton agar plates (Becton-Dickinson, Spain) for the following antimicrobials: imipenem, meropenem, ceftazidime, cefepime, cefotaxime, amikacin, tobramycin, gentamicin, kanamycin, tigecycline, ciprofloxacin, levofloxacin, ceftazidime-avibactam and fosfomycin. Susceptibility to colistin was assessed by gradient diffusion on Iso-Sensitest agar plates (ThermoFisher, United Kingdom). On note, colistin susceptibility was tested under research use only (RUO) acknowledgment since the recommended method for *in vitro* diagnostic (IVD) is broth microdilution (European Committee on Antimicrobial Susceptibility Testing (EUCAST), 2016). The MICs were interpreted according to EUCAST breakpoints for Enterobacterales (European Committee on Antimicrobial Susceptibility Testing (EUCAST), 2021). *Escherichia coli* ATCC 25922 was used for quality control.

Production of KPC, OXA-48-like, VIM, IMP or NDM carbapenemases was detected with the NG-Test^®^CARBA5 (NG-Biotech, France) and confirmed by PCR using previously described primers and conditions (Queenan and Bush, 2007; Solé et al., 2011; Bogaerts et al., 2013). Amplification products were purified from agarose gels and sent for Sanger sequencing (Genewiz, Germany) whenever necessary. The allelic identity of all genes was determined by sequence alignment with reference sequences retrieved from public repositories (PRJNA313047, last accessed June, 2021).

### Clonal Relatedness

Clonality was studied by pulsed-field gel electrophoresis (PFGE) using *Xba*I (New England BioLabs Inc., United States) genomic digestions and a CHEFF-DRIII system (Bio-Rad, Spain) (Marí-Almirall et al., 2021). Molecular patterns were analyzed with InfoQuest^TM^FP-v.5.4 (Bio-Rad, Spain) and the unweighted pair group method with arithmetic mean (UPGMA) to create dendrograms based on Dice’s similarity coefficient. Bandwidth tolerance and optimization values were set at 2% and isolates were considered within the same PFGE cluster (pulsotype) if their Dice similarity index was >85%.

Multi-locus sequence typing (MLST) was performed according to the Pasteur scheme for *K. pneumoniae* and the Achtman scheme for *E. coli* (Diancourt et al., 2005; Wirth et al., 2006).

### Plasmid Analysis

Conjugation assays were carried out using *E. coli* MC1061 resistant to rifampicin and sodium azide as the recipient strain. Transconjugant strains were selected in LB agar plates supplemented with 1 mg/L of meropenem and 100 mg/L of sodium azide (Sigma-Aldrich, Spain). The location of resistant genes was determined by S1-nuclease digestion (New England BioLabs Inc., United States) followed by PFGE and Southern blot hybridization with digoxigenin-labeled PCR-probes against *bla*_KPC_ (Bogaerts et al., 2013). Plasmid incompatibility groups were determined using the PBRT-2.0 kit (Diatheva, Italy) (Carattoli et al., 2005). The genetic environment of *bla*_KPC_ was determined by PCR using primers listed in Supplementary Table 1 and confirmed by long-read sequencing.

Genomic DNA was extracted using the Wizard Genomic DNA purification kit (Promega, Spain) and sequenced on a MinION instrument (Oxford-Nanopore, United Kingdom) using the rapid barcoding kit (SQK-RBK004) for library preparation and a R9.4.1 flowcell following the manufacturer’s instructions. Basecalling was done with Guppy-v3.0.3 and demultiplexing with qcat-v1.1.0^1^. FASTQ files were mapped using Minimap2-v2.17 against plasmids from Enterobacterales (Orlek et al., 2017). Mapping reads were assembled with Flye-v2.5^2^. Annotation was done with Prokka-v.1.12 combined with BLASTP/BLASTN searches against the UniProtKB/Swiss-Prot and RefSeq databases (Seemann, 2014).

ResFinder^3^, PlasmidFinder^4^, and ISFinder^5^ were used to identify antimicrobial resistance genes, plasmid replicons and mobile elements, respectively. The presence of virulence genes in plasmid sequences was checked against the Pasteur MLST site^6^. Genetic diagrams were drawn using SnapGene^®^Viewer-v5.1.2^7^ and CGViewAdvanced-v.0.0.1 (Stothard and Wishart, 2005).

FASTQ files of isolates MC-2-1, MC-2-207, MC-2-230, MC-2-240, MC-2-251, MC-2-304, MC-2-315, MC-2-316, MC-2-362, and MC-2-387, were deposited into the NCBI Sequence Read Archive under accession numbers SRX11379706, SRX11379707, SRX11379708, SRX11379709, SRX11379710, SRX11379711, SRX11379712, SRX11379713, SRX11379714, and SRX11379715, respectively; BioProject PRJNA744857.

## Results

### Bacterial Isolation and Clonal Relatedness

In mid-July 2018, five carbapenem-resistant *K. pneumoniae* isolates were recovered from surveillance samples (rectal swabs) upon the implementation of a “zero-resistance” surveillance program to screen all patients admitted to intensive care units within the hospital. All five isolates produced a KPC-type enzyme. During the following weeks, additional surveillance samples and an increasing number of clinical samples flagged positive for KPC-producing Enterobacterales. By the end of August, 87 KPC-producing isolates had been recovered and infection control measures were initiated, including exhaustive room cleaning twice a day, reinforcing surveillance measures, skin cleaning with 2% chlorhexidine wipes, and oral decolonization of patients (amikacin, colistin, nystatin) (Pellicé et al., 2021).

**FIGURE 1:**
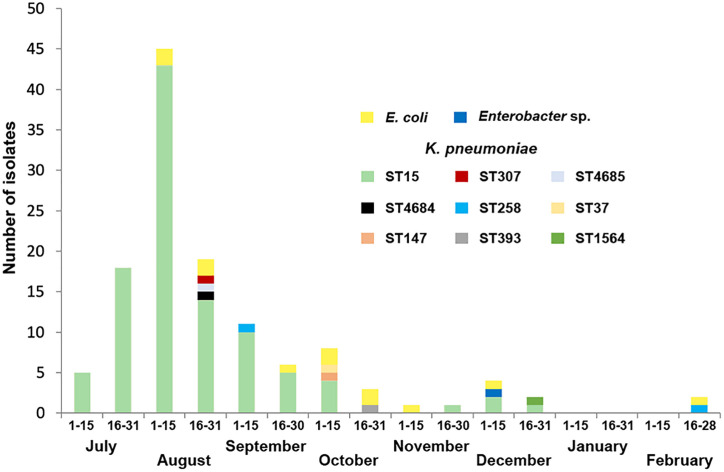
Temporal distribution of KPC-producing *K. pneumoniae* isolates according to sequence types (STs) as well as KPC-producing *E. coli* and *Enterobacter* sp. isolates recovered in this study.

During the following months the number of KPC-producing isolates rapidly decreased and the outbreak was considered eradicated by November 2018, although some isolates were still recovered up until February 2019 (Figure 1). Overall, 125 isolates (112 *K. pneumoniae*, 12 *E. coli*, and 1 *Enterobacter* sp.) from 101 patients were collected and included for further studies. Thirty-two isolates (25.6%) were detected from diagnosis samples and 93 (74.4%) were isolated from surveillance samples (Supplementary Table 2). The outbreak involved the screening of 2031 patients. The Index case could not be identified.

All *K. pneumoniae* strains and the single *Enterobacter* sp. isolate were classified into 11 PFGE clusters or pulsotypes (designated as PTk, to differentiate from PTc, derived from *E. coli* isolates, see below) with a major cluster, PTk1, that included 103 isolates while all other clusters just contained singletons. According to MLST, PTk-1 *K. pneumoniae* isolates were identified as ST15, PTk-2 as ST4684, PTk-3 as ST4685, PTk-5 as ST147, PTk-6 as ST1564, PTk-7 and PTk-8 as ST258, Ptk-9 as ST307, PTk-10 as ST393, PTk-11 as ST37, and PTk-4 corresponded to the single *Enterobacter* sp. isolate (Supplementary Figure 1).

Among *E. coli* isolates, PFGE showed higher clonal heterogeneity, since the 12 strains were grouped into 10 clusters (PTc 1-10), with only clusters 1 and 10 containing two strains each and all other pulsotypes corresponding to singletons (Supplementary Figure 2). According to MLST, the PTc-1 *E. coli* isolates were identified as ST101, PTc-2 and PTc-8 *E. coli* isolates were identified as ST410, PTc-3 as ST38, PTc-4 as ST8576, PTc-5 as ST1236, PTc-6 as ST1193, PTc-7 as ST131, PTc-9 as ST1431 and PTc-10 as ST1642.

One strain from each cluster of either *K. pneumoniae* or *E. coli* were selected for further phenotypic and genotypic characterization. A total of 24 isolates were eventually selected, 13 *K. pneumoniae*, 10 *E. coli* isolates, and 1 *Enterobacter* sp. (Table 1).

**TABLE 1:** Antimicrobial susceptibility and molecular characterization of representative KPC-producing *K. pneumoniae*, *Enterobacter* sp. and *E. coli* isolates.

Strains	Bacterial Species	*bla* gene	ST	MIC (mg/L)															Tn Type	P Inc	P Size
	
				IPM	MEM	CAZ	FEP	CTX	AMK	TOB	GEN	KAN	CST	TGC	CIP	LVX	CAZ/AVI	FOF			
MC-2-1	*K. pneumoniae*	KPC-2	ST15	24	>32	32	6	>32	1	0,38	0,38	1	2	1,5	12	4	0,75	24	pMC-2-1	**IncFIIk**	106 Kb
MC-2-16	*K. pneumoniae*	KPC-2	ST15	>32	>32	24	6	>32	1,5	0,5	0,38	1,5	0,094	1	>32	4	0,75	32	pMC-2-1	IncFIIk	106 Kb
MC-2-20	*K. pneumoniae*	KPC-2	ST15	>32	>32	24	4	>32	1	0,38	0,38	1,5	1	1,5	16	3	0,75	12	pMC-2-1	IncFIIk	106 Kb
MC-2-146	*K. pneumoniae*	KPC-2	ST15	>32	>32	32	48	>32	1	0,38	0,38	1	0,25	1,5	16	6	1	32	pMC-2-1	IncFIIk	106 Kb
MC-2-177	*E. coli*	KPC-2	ST410	4	3	192	32	>32	4	16	32	32	0,5	0,5	>32	>32	0,5	1	pMC-2-1	IncFIIk	106 Kb
MC-2-196	*E. coli*	KPC-2	ST8576	>32	16	48	>256	>32	1	0,38	0,5	1	0,125	0,75	0,008	0,023	0,125	1	pMC-2-1	IncFIIk	106 Kb
MC-2-203	*K. pneumoniae*	KPC-2	ST4684	4	>32	24	>256	>32	1	0,25	0,38	1	0,38	1	0,016	0,047	0,38	8	pMC-2-1	IncFIIk	106 Kb
MC-2-207	*E. coli*	KPC-2	ST1236	32	16	192	>256	>32	2	0,5	0,5	2	0,325	0,25	0,016	0,032	0,38	0,5	pMC-2-1	**IncFIIk**	106 Kb
MC-2-216	*K. pneumoniae*	KPC-2	ST4685	3	1	48	4	>32	1,5	2	0,38	4	0,25	1	0,5	0,38	0,25	6	pMC-2-1	IncFIIk	106 Kb
MC-2-230	*K. pneumoniae*	KPC-2	ST307	24	>32	24	8	>32	1	0,25	0,25	1	1	0,75	2	2	0,75	16	pMC-2-1	**IncFIIk IncN**	281 Kb
MC-2–240	*E. coli*	KPC-2	ST38	1	1	8	6	32	1,5	0,75	0,5	1,5	0,38	0,19	0,008	0,16	0,125	0,75	pMC-2-1	**IncFIIk**	190, 50 Kb
MC-2-251	*K. pneumoniae*	KPC-3	ST258	>32	>32	>256	>32	>32	32	16	1	>256	0,25	1	>32	>32	2	8	Tn*4401*	**IncFIIk**	78 Kb
MC-2-285	*E. coli*	KPC-2	ST410	12	8	24	8	>32	1	0,19	0,19	>256	0,25	0,25	>32	>32	0,19	>1024	pMC-2-1	IncFIIk	106 Kb
MC-2-303	*K. pneumoniae*	KPC-2	ST37	4	>32	24	12	>32	1	0,19	0,19	0,75	0,38	2	0,125	0,38	1	32	pMC-2-1	IncFIIk	106 Kb
MC-2-304	*E. coli*	KPC-2	ST1642	12	1,5	4	3	>32	4	16	128	>256	0,125	>32	>32	>32	0,19	0,75	pMC-2-1	**IncFIIk IncU**	170 Kb
MC-2-306	*K. pneumoniae*	KPC-2	ST147	>32	>32	16	8	>32	2	8	16	16	3	3	>32	>32	1	32	pMC-2-1	IncFIIk	106 Kb
MC-2-315	*E. coli*	KPC-2	ST101	>32	>32	192	128	>32	1,5	0,5	0,25	1,5	0,125	0,75	0,125	0,5	0,75	1	pMC-2-1	**IncFIIk**	101 Kb
MC-2-316	*K. pneumoniae*	KPC-2	ST393	>32	>32	16	16	>32	1,5	0,25	0,38	1	0,25	0,75	0,016	0,047	0,75	16	pMC-2-1	**IncFIIk**	109 Kb
MC-2-328	*E. coli*	KPC-2	ST1431	4	4	12	192	>32	1	0,5	0,25	2	0,25	0,5	>32	>32	0,125	0,75	pMC-2-1	IncFIIk	106 Kb
MC-2-350	*E. coli*	KPC-2	ST1193	3	24	16	24	>32	2	16	>256	16	0,125	0,38	>32	>32	0,25	0,75	pMC-2-1	IncFIIk	106 Kb
MC-2-362	*Enterobacter* sp.	KPC-2	ND	>32	>32	48	48	>32	2	12	32	16	1	4	>32	>32	1,5	12	pMC-2-1	**IncFIIk IncN IncU**	408 Kb
MC-2-382	*E. coli*	KPC-2	ST131	24	24	96	256	>32	6	16	16	16	1	0,25	>32	>32	0,25	1	pMC-2-1	IncFIIk	106 Kb
MC-2-387	*K. pneumoniae*	KPC-2,-3	ST258	>32	>32	>256	>32	>32	32	>256	16	>256	0,38	1,5	>32	>32	1	32	pMC-2-1 Tn*4401*	**IncFIIk**	120; 28 Kb
MC-22-164	*K. pneumoniae*	KPC-2	ST1564	>32	>32	24	64	>32	1	0,19	0,38	1	1	2	1,5	3	1	>1024	pMC-2-1	IncFIIk	106 Kb

*ST, sequence type; IPM, imipenem; MEM, meropenem; CAZ, ceftazidime; FEP, cefepime; CTX, cefotaxime; AMK: amikacin; TOB: tobramycin; GEN, gentamicin; KAN, kanamycin; CST, colistin; TGC, tigecycline; CIP, ciprofloxacin; LVX, levofloxacin; CAZ/AVI, ceftazidime/avibactam; FOF, fosfomycin; Tn Type, type of transposon containing the *bla*_KPC_ gene; P Inc, Incompatibility group of plasmids containing the *bla*_KPC_ gene. Inc groups from sequenced plasmids are shown in bold face; P Size, Size of plasmids containing the *bla*_KPC_ gene; ND, not determined.*

### Phenotypic and Molecular Characterization of Resistance

The MICs of selected isolates are shown in Table 1. All isolates were non-susceptible to cephalosporins and carbapenems, but were susceptible to ceftazidime-avibactam, fosfomycin and colistin, except for MC-2-285 and MC-22-164, that were highly resistant to fosfomycin.

Sanger sequencing identified the *bla*_KPC–2_ variant in all isolates except for the MC-2-251 and MC-2-387 isolates, that carried *bla*_KPC–3_ (note that MC-2-387 also carried *bla*_KPC–2_). The genes for CTX-M-group 1 enzymes were also detected in 7 of the 24 strains, while CTX-M-group 2, 9, and 25 were only found in a few strains (Supplementary Table 2). CTX-M-group 8 was not detected. All isolates were negative for the presence of *bla*_OXA–48_, *bla*_NDM_, *bla*_VIM_, or *bla*_IMP_.

**FIGURE 2:**
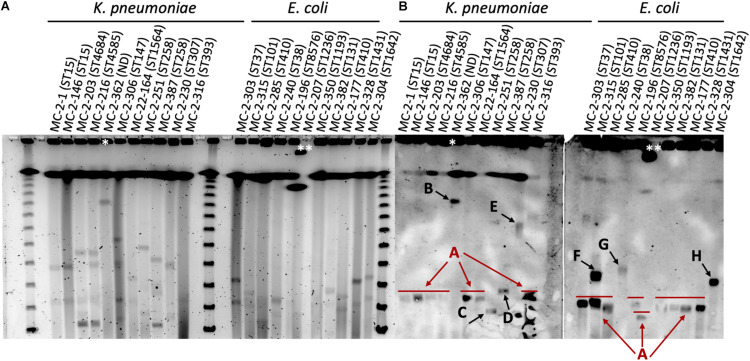
**(A)** S1-nuclease-PFGE profiles and **(B)** corresponding southern hybridization membranes with *bla*_KPC_ probes of selected *K. pneumoniae* and *E. coli* isolates in this study. The ST of both *K. pneumoniae* and *E. coli* isolates is shown in brackets. Arrows indicate the different arbitrarily designated plasmid types. Lambda ladder PFGE Marker (New England Biolabs, United States). *Enterobacter sp. isolate. **The PFGE plug moved from the loading site, so the band is shifted. ND, not determined.

S1-nuclease-PFGE profiles of selected strains revealed the presence of different plasmids (according to size) that contained the *bla*_KPC_ gene. Eight of the selected *K. pneumoniae* strains harbored *bla*_KPC–2_ within a plasmid of circa 110 kb in size (plasmid type A, Figure 2), including the ST15 strains. The two ST258 strains MC-2-251 and MC-2-387 carrying *bla*_KPC–3_, however, showed hybridization signals with plasmids of circa 80 Kb and 120 Kb, respectively (plasmid types C and D, Figure 2), and the single ST307 strain (MC-2-230) carried *bla*_KPC–2_ within a plasmid of circa 300 kb (plasmid type E, Figure 2). Likewise, the *Enterobacter* sp. isolate harbored *bla*_KPC–2_ in a plasmid of approximately 400 Kb (plasmid type B, Figure 2).

Among selected *E. coli* isolates, eight isolates also carried *bla*_KPC–2_ in plasmids of similar size as that of type A plasmids in ST15 *K. pneumoniae* (110 Kb), although the MC-2-315 isolate presented a second hybridization signal at circa 180 Kb (plasmid type F, Figure 2). Two additional strains, MC-2-240 and MC-2-304, carried *bla*_KPC–2_ in plasmids of different sizes. MC-2-240 showed a hybridization band at approximately 190 Kb, and MC-2-304 at 170 Kb (plasmid types G and H, respectively, Figure 2).

Notably, all *E. coli* isolates but one and the single *Enterobacter* sp. isolate recovered during the outbreak, originated from patients that previously carried a ST15 KPC-2-producing *K. pneumoniae*. On the other hand, only three out of the nine non-ST15 *K. pneumoniae* isolates were recovered from patients also co-carrying a ST15 isolate (Supplementary Figure 4 and Supplementary Table 2). Plasmid replicon typing identified the presence of multiple plasmids in some of the strains (Table 1), but all strains were positive for a replicon belonging to the IncFIIk incompatibility group. Conjugation assays using the ST15 isolate MC-2-1 as donor and the *E. coli* strain MC1061 as recipient, showed that the acquisition of a *bla*_KPC–2_ gene was associated with the transfer of an IncFIIk plasmid (not shown).

### Plasmid Sequencing

**FIGURE 3:**
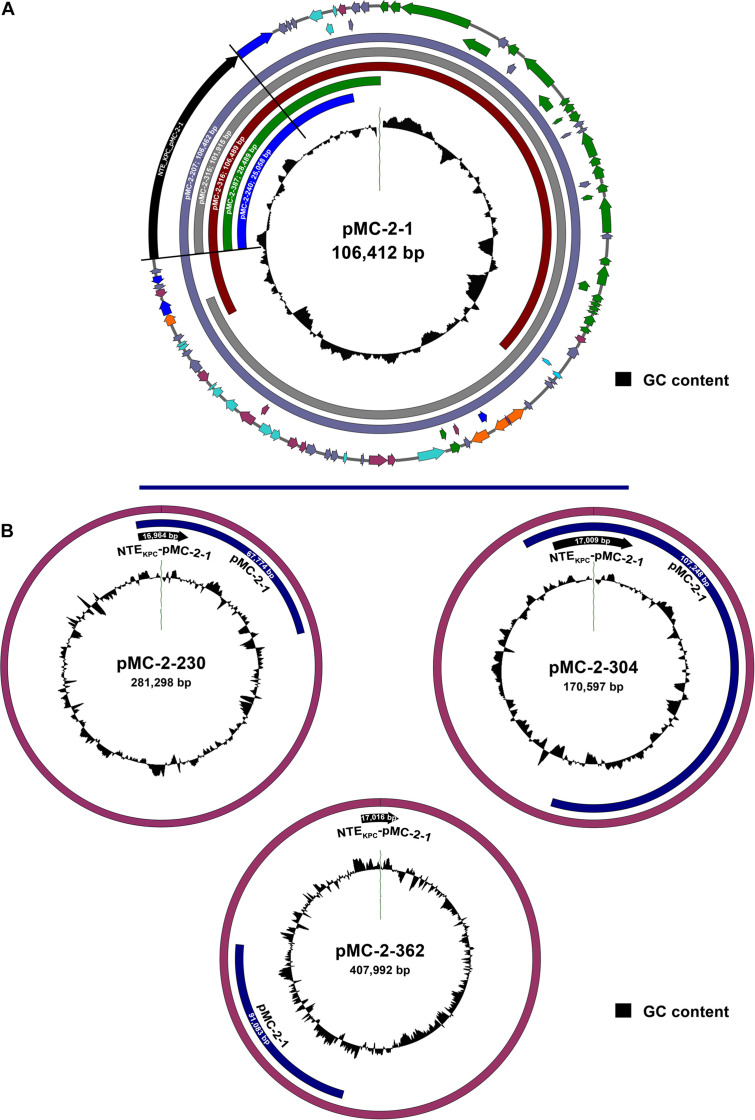
**(A)** Schematic representation of the 106,412 bp IncFIIk plasmid from the ST15 *K. pneumoniae* strain MC-2-1 (pMC-2-1) carrying *bla*_KPC–2_ within a NTE_KPC_-pMC-2-1 genetic structure and sequence alignment with similar IncFIIk plasmid backbones from strains MC-2-207, MC-2-315, MC-2-316, MC-2-387, and MC-2-240 (only the aligned region is shown). Arrows are proportional to the lengths of the genes and oriented in the direction of transcription. Red arrows represent resistance genes, orange arrows represent full-length transposon-related genes and ISs, dark blue arrows represent partial or truncated transposon-related genes and ISs, green arrows indicate genes involved in plasmid conjugation and light blue arrows shown genes related to plasmid replication/maintenance. Blue-gray arrows show putative or hypothetical genes and plum arrows show genes involved in other functions. The inner circle shows the GC content. The fully annotated sequence of plasmid pMC-2-1 is shown in Supplementary Figure 3. **(B)** Schematic representation of the larger IncFIIk plasmids carrying *bla*_KPC–2_ recovered from strains MC-2-230, MC-2-304, and MC-2-363. The location of the genetic structure NTE_KPC_-pMC-2-1 harboring *bla*_KPC–2_ is shown as well as the region matching the pMC-2-1 sequence. The inner circle shows the GC content.

Ten isolates, 5 *K. pneumoniae*, 4 *E. coli*, and 1 *Enterobacter* sp., representative of strains carrying all the different plasmid types identified with hybridization probes, were further selected for long-read plasmid sequencing. Plasmid sequencing identified an IncFIIk plasmid of 106,412 bp carrying *bla*_KPC–2_ in the ST15 *K. pneumoniae* MC-2-1 isolate, designated as pMC-2-1 (Supplementary Figure 3). The *bla*_KPC–2_ gene was the only antibiotic resistance gene present in the pMC-2-1 plasmid that also harbored several genes involved in conjugative transfer. The presence of plasmid-associated virulence genes was not detected in plasmid pMC-2-1 (Supplementary Figure 3). An almost identical plasmid of 106,462 bp was found in the *E. coli* strain MC-2-207, and highly similar IncFIIk plasmids of 109,489 and 101,915 bp were located in the *K. pneumoniae* and *E. coli* isolates MC-2-316 and MC-2-315, respectively, both recovered from the same patient (Figure 3A), in good agreement with the carriage of *bla*_KPC–2_ in a plasmid of circa 110 Kb (plasmid type A), as identified by S1-digestion. Unfortunately, plasmid type F of circa 180 Kb, also present in strain MC-2-315 according to S1-digestion, could not be identified by long-read sequencing analysis.

The *K. pneumoniae* isolate MC-2-230, the *Enterobacter* sp. isolate MC-2-362 and the *E. coli* isolate MC-2-304, carried *bla*_KPC–2_ within IncFIIk plasmids of 281,298, 407,992, and 170,594 bp, respectively, much larger than that of ST15 strains and in good agreement with results from S1-nuclease digestion. Additional replicon types from IncN and/or IncU incompatibility groups were also identified in these plasmids. Interestingly, the entire 106 Kb sequence that made up for the pMC-2-1 plasmid was also found inserted within the plasmids harboring KPC in strains MC-2-304 and MC-2-362, and the 281 Kb plasmid from strain MC-2-230 also carried a 67 kb fragment from pMC-2-1 (Figure 3B). On the other hand, the two ST258 *K. pneumoniae* isolates (MC-2-251 and MC-2-387) harbored *bla*_KPC–3_ within IncFIIk plasmids of 78,515 and 120,395 bp, and there was no similarity at all between these two plasmids and those of ST15 strains as there was no resemblance between them either.

In the *E. coli* strain MC-2-240 we only detected *bla*_KPC–2_ within a 25,058 bp plasmid showing 100% similarity with pMC-2-1, and a similar plasmid of 28,498 bp was also detected in strain MC-2-387, which also carried *bla*_KPC–3_ in the 120,395 bp plasmid (Figure 3A).

**FIGURE 4:**
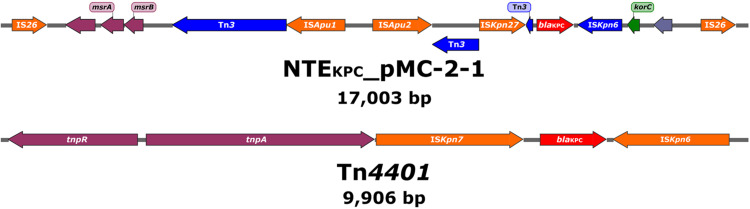
Schematic drawing showing the genetic elements surrounding the *bla*_KPC_ genes in the NTE_KPC_-pMC-2-1 variant and Tn*4401*, respectively. Arrows are oriented in the direction of transcription. Red arrows show the *bla*_KPC_ gene, orange arrows represent full-length insertion sequences (IS), dark blue arrows represent partial or truncated transposon-related regions and ISs, and green arrows indicate genes involved in gene regulation. Blue-gray arrows show putative or hypothetical genes and plum arrows show genes involved in other functions.

Notably, in all plasmids *bla*_KPC–2_ was not located within the canonical Tn*4401* element but inside an IS26-based composite transposon of roughly 17 Kb containing an IS*Kpn27*-*bla*_KPC–2_-ΔIS*Kpn6*-*korC* core structure as well as Tn*3*-associated sequences upstream from IS*Kpn27* (Figure 4). This rearrangement was tentatively designated as NTE_KPC_-pMC-2-1. The genetic structures surrounding the *bla*_KPC–3_ gene in both ST258 strains, however, did match that of a classical Tn*4401* element (Figure 4). Specific primers to amplify the structures associated with NTE_KPC_-pMC-2-1 were designed (Supplementary Table 1 and Supplementary Figure 5) and used to verify the carriage of either the canonical Tn*4401* transposon or the NTE_KPC_-pMC-2-1 variant in all 125 isolates of the outbreak. Interestingly, NTE_KPC_-pMC-2-1 was identified in all isolates but in the ST258 *K. pneumoniae* isolate MC-2-251.

In addition, all plasmid sequences were checked for the presence of plasmid-mediated virulence genes producing salmochelin, aerobactin, the hypermucoviscosity factor and/or other virulence genes (Russo et al., 2018), but none of the plasmid sequences carrying *bla*_KPC_ harbored any known virulence determinant.

## Discussion

The emergence of KPC-producing Enterobacterales has been increasing worldwide during the last decades (Lee et al., 2019). Since its first description in 1996 in the United States, KPC has spread through the globe, becoming endemic in areas such as the United States, Israel, Greece, and Italy, and sporadic spread being reported in additional European countries as well as in the Asia-Pacific region (Lee et al., 2019). Many of the descriptions from European countries have been related to patients traveling to endemic areas.

In Spain, the first KPC report dates back to 2009 and it was associated with the rapid spread of a ST384 *K. pneumoniae* strain carrying *bla*_KPC–3_ within a Tn*4401a* structure (Curiao et al., 2010). KPC-2 and KPC-3-producing Enterobacterales were later reported in the central region of Spain and Andalusia mainly associated with *K. pneumoniae* isolates belonging to the CG258 or ST101 (López-Cerero et al., 2014; Porres-Osante et al., 2014; Oteo et al., 2016). In Catalonia, a single KPC-3-producing *K. pneumoniae* isolate belonging to ST258 was recovered from a human patient in 2015 (Piedra-Carrasco et al., 2018) but, to our knowledge, our study constitutes the first hospital outbreak caused by KPC-producing Enterobacterales in this region.

Here we report an outbreak caused by KPC-producing *K. pneumoniae* and *E. coli* in a tertiary hospital in Barcelona that initiated during the summer of 2018. It involved 125 Enterobacterales isolates recovered from surveillance (74.4%) or diagnosis (25.6%) samples. Outbreak identification was possible upon the implementation of an active surveillance program in ICU patients, but it is likely that KPC-producing *K. pneumoniae* isolates had already been circulating in asymptomatic fecal carriers for some time. The outbreak was caused by the rapid spread of a carbapenem-resistant ST15 *K. pneumoniae* strain carrying *bla*_KPC–2_ in an IncFIIk plasmid of approximately 106 Kb and located within a non-Tn*4401* genetic element (NTE_KPC_-pMC-2-1).

Nevertheless, upon the initial stage of clonal spread, we speculate that the IncFIIk plasmid was successfully transferred first to *E. coli* isolates in patients either infected or colonized with the original ST15 strain, but later to other strains of *K. pneumoniae* or even *Enterobacter* spp. (Figure 1 and Supplementary Figure 4). This is supported by the fact that in at least 14 instances the same patient co-carried a *bla*_KPC–2_-ST15 *K. pneumoniae* strain together with either a *bla*_KPC–2_-*K. pneumoniae* belonging to a different sequence type or a *bla*_KPC–2_-*E. coli* or *Enterobacter* sp. strain, but also because all plasmids carrying *bla*_KPC–2_ in *K. pneumoniae* or *E. coli* strains shared high similarity with the IncFIIk plasmids from the ST15 *K. pneumoniae* strains, including the NTE_KPC_-pMC-2-1 structure surrounding *bla*_KPC–2_. Some genetic rearrangements, however, seem to have occurred, mainly in non-*K. pneumoniae* strains where the 106 Kb IncFIIk plasmid either co-integrated with other plasmids or suffered extensive genetic reduction. Therefore, intra- and inter-species dissemination of resistance also contributed to the spread of the outbreak. KPC transfer to other *K. pneumoniae* strains was not as heavily associated with co-carriage within the same patient, as opposite to inter-species dissemination, but such finding may also reflect a selection bias in the microbiology laboratory. Interestingly though, the clonal dissemination of non-ST15 strains was not detected. Recently, San Millán and co-workers proposed a similar hospital transmission dynamic for pOXA-48, where patient-to-patient transmission was tightly associated with the dissemination of a particular high-risk clone, while intra- and inter-species transmission of the plasmid was linked to concurrent gut colonization (León-Sampedro et al., 2021). As in the case of OXA-48, this genetic exchange represents an opportunity for the resistance gene to rearrange and shuffle into new plasmids and/or hosts, some of which may become more successful (Millan, 2018).

The genetic structures associated with the core structure of NTE_KPC_-pMC-2-1 identified in this work (IS*Kpn27*-*bla*_KPC–2_-ΔIS*Kpn6*-*korC*) had already been reported by other studies and seem to constitute a common rearrangement associated with *bla*_KPC–2_ mostly in isolates from China (Wang et al., 2015; Zhang et al., 2017). In Spain though, this structure has also been identified in IncP-6 plasmids from *Citrobacter freundii*, *Enterobacter cloacae*, and *Klebsiella oxytoca* but, to our knowledge, have never been associated with *K. pneumoniae* isolates of human origin, most likely suggesting a recent acquisition (Yao et al., 2017; Pérez-Vazquez et al., 2019).

Two sporadic *K. pneumoniae* strains belonging to the clonal group CG258 and carrying the *bla*_KPC–3_ gene within a canonical Tn*4401* in two different non-related plasmids were also detected in the study and, most likely, constituted an independent event unrelated to the spread of the outbreak. Nevertheless, one of such strains also managed to acquire a partial plasmid sequence containing the entire NTE_KPC_-pMC-2-1 element.

The identification of a carbapenem resistant ST307 *K. pneumoniae* isolate in this study is also worth mentioning, as strains from this epidemic clonal group have recently been responsible for a hospital outbreak in Germany associated with multidrug resistance but also with the hypervirulent *Klebsiella pneumoniae* phenotype (hvKp). The hvKp phenotype was identified among ST307 isolates upon the acquisition of several plasmid-mediated virulence genes that merged with a resistance plasmid, hence creating a *mosaic* plasmid carrying both resistance and virulence genes (Heiden et al., 2020). In our study the ST307 strain (MC-2-230) carried *bla*_KPC–2_ in a plasmid of 280 Kb also likely resulting from the merging of two different plasmids and, hence, the potential carriage of plasmid-mediated virulence genes was investigated. Fortunately, none of the plasmid-mediated virulence factors associated with the hvKp phenotype were present in plasmid pMC-2-230 nor in any other plasmid sequenced in this study. Likewise, none of the *K. pneumoniae* strains showed a hypermucoid phenotype, which is characteristic (although not exclusive) of hvKp (Lan et al., 2021).

We acknowledge several limitations in our study. First, WGS was performed under a single long-read sequencing approach, and we acknowledge that the use of a hybrid approach would have allowed for additional and more accurate comparisons. In addition, only a selected group of isolates were sequenced and sequence similarity was, therefore, assumed for the remaining isolates on the basis of PFGE, MLST and conventional PCR data. Unfortunately, further WGS analyses were beyond our possibilities but we expect that results from this study will contribute to a better implementation of WGS pipelines in our institution.

## Conclusion

We report a hospital outbreak caused by the clonal dissemination of KPC-producing ST15 *K. pneumoniae* mainly among colonized carriers but also by the intra- and inter-species transmission of the *bla*_KPC–2_ gene associated with plasmid conjugation and/or transposon dissemination. The ST15 clonal lineage is considered a high-risk clone and has been associated with KPC-2-producing isolates in Bulgaria and Vietnam (Markovska et al., 2015; Berglund et al., 2019), and it has also been reported in Portugal and Italy (Rodrigues et al., 2016; Fasciana et al., 2019). In Spain, ST15 has been reported but only associated with the production of OXA-48 (Madueño et al., 2017). Nevertheless, ST15 has been attributed with a high potential for horizontal gene acquisition and dissemination and it is critical that active surveillance strategies are prolonged over time to allow for the rapid detection and eradication of these highly resistant and virulent clones (Berglund et al., 2019).

## MERCyCAT Study Group

Pepa Pérez Jove, Emma Padilla, and Mónica Ballestero-Téllez (Catlab, Centre Analítiques Terrassa AIE), Yuliya Zboromyrska, Miguel Ángel Benítez, Raquel Clivillé, Sabina González, and Iolanda Calvet (Consorci del Laboratori Intercomarcal de l’Alt Pendès, l’Anoia i el Garraf), Carmen Gallés (Corporació de Salut del Maresme i la Selva), Goretti Sauca (Hospital de Mataró), Carmina Martí-Sala and M^a^ Angeles Pulido (Hospital General de Granollers), Anna Vilamala (Hospital General de Vic), Araceli González-Cuevas (Hospital General del Parc Sanitari Sant Joan de Déu), Amadeu Gené (Hospital Sant Joan de Déu de Barcelona), Gloria Trujillo and Joan Lopez Madueño (Hospital Sant Joan de Déu de Manresa), Xavier Raga (Hospital Sant *Pau*I Santa Tecla), Frederic Gómez, Ester Picó, and Carolina Sarvisé (Hospital Universitari Joan XXIII de Tarragona), Isabel Pujol and Xesca Font (Hospital Universitari Sant Joan de Reus).

## Data Availability Statement

The datasets presented in this study can be found in online repositories. The names of the repository/repositories and accession number(s) can be found in the article/Supplementary Material.

## Author Contributions

MM-A, NF, and MF contributed to the conception, design, and implementation of the study, acquisition of laboratory and clinical data, analysis of the results, drafting the manuscript, and approval of the final version of the manuscript. CC, JViñ, ER, AC, and LM contributed to the acquisition of laboratory data, analysis of the results, and review and approval of the final version of the manuscript. MP, AV, IC, LR-S, GS, and AD contributed to the design of the study, acquisition of clinical data, analysis of the results, and review and approval of the final version of the manuscript. OF, PC, FB, FM, JM, and ÁS contributed to the design of the study, analysis of the results, and review and approval of the final version of the manuscript. CP, JVil, and IR contributed to the conception, design, and implementation of the study, analysis of the results, drafting the manuscript, and approval of the final version of the manuscript. All authors critically revised the manuscript for intellectual content and read and approved the final manuscript.

## Conflict of Interest

The authors declare that the research was conducted in the absence of any commercial or financial relationships that could be construed as a potential conflict of interest.

## Publisher’s Note

All claims expressed in this article are solely those of the authors and do not necessarily represent those of their affiliated organizations, or those of the publisher, the editors and the reviewers. Any product that may be evaluated in this article, or claim that may be made by its manufacturer, is not guaranteed or endorsed by the publisher.

## Footnotes

1
https://github.com/nanoporetech/qcat

2
https://github.com/fenderglass/Flye

3
https://cge.cbs.dtu.dk/services/ResFinder/

4
https://cge.cbs.dtu.dk/services/PlasmidFinder/

5
https://www-is.biotoul.fr/

6
https://bigsdb.web.pasteur.fr/

7
https://www.snapgene.com/
