# Supplementary material for: Clonal Spread and Intra- and Inter-Species Plasmid Dissemination Associated With Klebsiella pneumoniae Carbapenemase-Producing Enterobacterales During a Hospital Outbreak in Barcelona, Spain
Source: Front Microbiol. 2021 Nov 18;12:781127. doi: 10.3389/fmicb.2021.781127 (PMC8637019; doi:10.3389/fmicb.2021.781127)
Supplement: Supplementary file 1 [file Data_Sheet_1.PDF]

Table S1. Primer sequences used to investigate genetic structures surrounding *bla*$_{KPC}$ genes.

#	Primer Name	Sequence 5'→3'	Reference
1	KPC-Out-F	CCTAACAAGGATGACAAGCACAG	This study
2	KPC-Out-R	CTAGACGGCGATACAGTGACA	This study
3	ISApu2-F	CAATGCAACCCAGTACCTCAAGC	This study
4	ISApu2-R	CCGTCTAACGTGTTGTTTTGC	This study
5	TnpA-Tn2-F	CACGATACTGATAGTGCTGACG	This study
6	TnpA-Tn2-IntR	GCAATACTGAGCTGATGAGC	This study
7	TnpA-Tn2-IntF	GCTCATCAGCTCAGTATTGC	This study
8	ISKpn27-F	TGTCTGGACTCGTGGGATCATG	This study
9	ISKpn27-R	TGTCAAGACCCGGCTGGTTATAC	This study
10	IS26-F	GGCACTGTTGCAAAGTTAGCG	This study
11	IS26-R	GGCACTGTTGCAAATAGTCGG	This study
12	KPC_4714	GAAGATGCCAAGGTCAATGC	Naas, 2008
13	KPC_4281	GGCACGGCAAATGACTA	Naas, 2008
14	Tn4401_IRL	CCAGAAAATTCCGTCATTCCG	Naas, 2008
15	Tn4401_IRR	GAAAATTCCGCCATTCCGC	Naas, 2008
16	KPC_Riout	ACGACCACGCACGCACAAAC	Naas, 2008
17	KPC_3781L	GCTTTCTTGCTGCCGCTGTG	Naas, 2008
18	KPC_3098_U	TGACCCTGAGCGGCGAAAGC	Naas, 2008
19	KPC_905L	GCGACCGGTCAGTTCCTTCT	Naas, 2008
20	KPC_816U	CACCTACACCACGACGAACC	Naas, 2008
21	KPC_141R3	TCACCGGCCCTCACCTTTGG	Naas, 2008

Naas T, Cuzon G, Villegas MV, Lartigue M-F, Quinn JP, Nordmann P. Genetic structures at the origin of acquisition of the beta-lactamase *bla*$_{KPC}$ gene. Antimicrobial agents and chemotherapy. 2008 Apr;52(4):1257–63.
