# Supplementary material for: Clonal Spread and Intra- and Inter-Species Plasmid Dissemination Associated With Klebsiella pneumoniae Carbapenemase-Producing Enterobacterales During a Hospital Outbreak in Barcelona, Spain
Source: Front Microbiol. 2021 Nov 18;12:781127. doi: 10.3389/fmicb.2021.781127 (PMC8637019; doi:10.3389/fmicb.2021.781127)
Supplement: Supplementary file 2 [file Data_Sheet_2.PDF]

Table S2. Microbiological and clinical features of KPC-producing Enterobacterales included in this study. Isolates are shown in chronological order.

Strain	Patient	Date	Species	SampleSource	SampleType	bla_{KPC}	$bla_{CTX.M}$	PT	ST	Plasmid	NTE_{KPC}
MC-2-3	Patient1	18/07/10	*K. pneumoniae*	Rectal swab	Surveillance	KPC-2	-	1	ST15	A	Positive
MC-2-6	Patient2	18/07/11	*K. pneumoniae*	Rectal swab	Surveillance	KPC-2	-	1	ST15	A	Positive
MC-2-5	Patient3	18/07/11	*K. pneumoniae*	Rectal swab	Surveillance	KPC-2	-	1	ST15	A	Positive
MC-2-4	Patient4	18/07/11	*K. pneumoniae*	Rectal swab	Surveillance	KPC-2	-	1	ST15	A	Positive
MC-2-7	Patient5	18/07/13	*K. pneumoniae*	Rectal swab	Surveillance	KPC-2	-	1	ST15	A	Positive
MC-2-8	Patient6	18/07/17	*K. pneumoniae*	Rectal swab	Surveillance	KPC-2	-	1	ST15	A	Positive
MC-2-10	Patient7	18/07/18	*K. pneumoniae*	Rectal swab	Surveillance	KPC-2	-	1	ST15	A	Positive
MC-2-9	Patient8	18/07/18	*K. pneumoniae*	Rectal swab	Surveillance	KPC-2	-	1	ST15	A	Positive
MC-2-11	Patient9	18/07/20	*K. pneumoniae*	Urine	Diagnosis	KPC-2	-	1	ST15	A	Positive
MC-2-12	Patient10	18/07/23	*K. pneumoniae*	Urine	Diagnosis	KPC-2	-	1	ST15	A	Positive
MC-2-16	Patient11	18/07/24	*K. pneumoniae*	Blood	Diagnosis	KPC-2	-	1	ST15	A	Positive
MC-2-13	Patient12	18/07/24	*K. pneumoniae*	Rectal swab	Surveillance	KPC-2	-	1	ST15	A	Positive
MC-2-17	Patient14	18/07/24	*K. pneumoniae*	Ascitic fluid	Surveillance	KPC-2	-	1	ST15	A	Positive
MC-2-1	Patient13	18/07/24	*K. pneumoniae*	Rectal swab	Surveillance	KPC-2	-	1	ST15	A	Positive
MC-2-18	Patient16	18/07/25	*K. pneumoniae*	Urine	Diagnosis	KPC-2	-	1	ST15	A	Positive
MC-2-19	Patient15	18/07/25	*K. pneumoniae*	Urine	Diagnosis	KPC-2	-	1	ST15	A	Positive
MC-2-15	Patient17	18/07/25	*K. pneumoniae*	Rectal swab	Surveillance	KPC-2	-	1	ST15	A	Positive
MC-2-14	Patient18	18/07/25	*K. pneumoniae*	Rectal swab	Surveillance	KPC-2	-	1	ST15	A	Positive
MC-2-20	Patient11	18/07/27	*K. pneumoniae*	Rectal swab	Surveillance	KPC-2	-	1	ST15	A	Positive
MC-2-21	Patient19	18/07/30	*K. pneumoniae*	Blood	Diagnosis	KPC-2	-	1	ST15	A	Positive
MC-2-146	Patient12	18/07/31	*K. pneumoniae*	Rectal swab	Surveillance	KPC-2	-	1	ST15	A	Positive

Strain	Patient	Date	Species	SampleSource	SampleType	*bla*KPC	*bla*CTX.M	PT	ST	Plasmid	NTEKPC
MC-2-22	Patient15	18/07/31	*K. pneumoniae*	Rectal swab	Surveillance	KPC-2	-	1	ST15	A	Positive
MC-2-23	Patient20	18/07/31	*K. pneumoniae*	Rectal swab	Surveillance	KPC-2	-	1	ST15	A	Positive
MC-2-26	Patient21	18/08/01	*K. pneumoniae*	Rectal swab	Surveillance	KPC-2	-	1	ST15	A	Positive
MC-2-24	Patient22	18/08/01	*K. pneumoniae*	Rectal swab	Surveillance	KPC-2	-	1	ST15	A	Positive
MC-2-148	Patient12	18/08/01	*K. pneumoniae*	Rectal swab	Surveillance	KPC-2	-	1	ST15	A	Positive
MC-2-25	Patient23	18/08/01	*K. pneumoniae*	Rectal swab	Surveillance	KPC-2	-	1	ST15	A	Positive
MC-2-151	Patient24	18/08/02	*K. pneumoniae*	Rectal swab	Surveillance	KPC-2	-	1	ST15	A	Positive
MC-2-149	Patient25	18/08/02	*K. pneumoniae*	Rectal swab	Surveillance	KPC-2	-	1	ST15	A	Positive
MC-2-150	Patient26	18/08/02	*K. pneumoniae*	Rectal swab	Surveillance	KPC-2	-	1	ST15	A	Positive
MC-2-153	Patient27	18/08/02	*K. pneumoniae*	Rectal swab	Surveillance	KPC-2	-	1	ST15	A	Positive
MC-2-155	Patient28	18/08/02	*K. pneumoniae*	Urine	Diagnosis	KPC-2	-	1	ST15	A	Positive
MC-2-152	Patient29	18/08/02	*K. pneumoniae*	Rectal swab	Surveillance	KPC-2	-	1	ST15	A	Positive
MC-2-156	Patient30	18/08/03	*K. pneumoniae*	Rectal swab	Surveillance	KPC-2	-	1	ST15	A	Positive
MC-2-157	Patient31	18/08/03	*K. pneumoniae*	Rectal swab	Surveillance	KPC-2	-	1	ST15	A	Positive
MC-2-159	Patient32	18/08/03	*K. pneumoniae*	Osteoarticular swab	Diagnosis	KPC-2	-	1	ST15	A	Positive
MC-2-160	Patient33	18/08/04	*K. pneumoniae*	Urine	Diagnosis	KPC-2	-	1	ST15	A	Positive
MC-2-161	Patient34	18/08/05	*K. pneumoniae*	Urine	Diagnosis	KPC-2	-	1	ST15	A	Positive
MC-2-168	Patient35	18/08/06	*K. pneumoniae*	Urine	Diagnosis	KPC-2	-	1	ST15	A	Positive
MC-2-166	Patient36	18/08/06	*K. pneumoniae*	Blood	Diagnosis	KPC-2	-	1	ST15	A	Positive
MC-2-163	Patient37	18/08/06	*K. pneumoniae*	Rectal swab	Surveillance	KPC-2	-	1	ST15	A	Positive
MC-2-169	Patient38	18/08/06	*K. pneumoniae*	Rectal swab	Surveillance	KPC-2	-	1	ST15	A	Positive
MC-2-164	Patient39	18/08/06	*K. pneumoniae*	Rectal swab	Surveillance	KPC-2	-	1	ST15	A	Positive
MC-2-162	Patient40	18/08/06	*K. pneumoniae*	Blood	Diagnosis	KPC-2	-	1	ST15	A	Positive
MC-2-167	Patient41	18/08/06	*K. pneumoniae*	Rectal swab	Surveillance	KPC-2	-	1	ST15	A	Positive
MC-2-170	Patient43	18/08/07	*K. pneumoniae*	Rectal swab	Surveillance	KPC-2	-	1	ST15	A	Positive

Strain	Patient	Date	Species	SampleSource	SampleType	***bla***KPC	***bla***CTX.M	PT	ST	Plasmid	NTEKPC
MC-2-177	Patient43	18/08/07	*E. coli*	Rectal swab	Surveillance	KPC-2	CTX-M-1	8	ST410	A	Positive
MC-2-171	Patient45	18/08/07	*K. pneumoniae*	Rectal swab	Surveillance	KPC-2	-	1	ST15	A	Positive
MC-2-176	Patient1	18/08/07	*K. pneumoniae*	Urine	Diagnosis	KPC-2	-	1	ST15	A	Positive
MC-2-175	Patient46	18/08/07	*K. pneumoniae*	Rectal swab	Surveillance	KPC-2	-	1	ST15	A	Positive
MC-2-172	Patient43	18/08/07	*K. pneumoniae*	Rectal swab	Surveillance	KPC-2	-	1	ST15	A	Positive
MC-2-174	Patient44	18/08/07	*K. pneumoniae*	Rectal swab	Surveillance	KPC-2	-	1	ST15	A	Positive
MC-2-173	Patient42	18/08/07	*K. pneumoniae*	Rectal swab	Surveillance	KPC-2	-	1	ST15	A	Positive
MC-2-178	Patient49	18/08/09	*K. pneumoniae*	Rectal swab	Surveillance	KPC-2	-	1	ST15	A	Positive
MC-2-183	Patient48	18/08/09	*K. pneumoniae*	Urine	Diagnosis	KPC-2	-	1	ST15	A	Positive
MC-2-179	Patient50	18/08/09	*K. pneumoniae*	Rectal swab	Surveillance	KPC-2	-	1	ST15	A	Positive
MC-2-181	Patient51	18/08/09	*K. pneumoniae*	Rectal swab	Surveillance	KPC-2	-	1	ST15	A	Positive
MC-2-189	Patient52	18/08/10	*K. pneumoniae*	Rectal swab	Surveillance	KPC-2	-	1	ST15	A	Positive
MC-2-188	Patient53	18/08/10	*K. pneumoniae*	Rectal swab	Surveillance	KPC-2	-	1	ST15	A	Positive
MC-2-191	Patient54	18/08/12	*K. pneumoniae*	Endometrial aspirate	Diagnosis	KPC-2	-	1	ST15	A	Positive
MC-2-190	Patient55	18/08/12	*K. pneumoniae*	Rectal swab	Surveillance	KPC-2	-	1	ST15	A	Positive
MC-2-192	Patient56	18/08/12	*K. pneumoniae*	Blood	Diagnosis	KPC-2	-	1	ST15	A	Positive
MC-2-193	Patient58	18/08/13	*K. pneumoniae*	Rectal swab	Surveillance	KPC-2	-	1	ST15	A	Positive
MC-2-195	Patient59	18/08/13	*K. pneumoniae*	Rectal swab	Surveillance	KPC-2	-	1	ST15	A	Positive
MC-2-196	Patient54	18/08/13	*E. coli*	Rectal swab	Surveillance	KPC-2	CTX-M-2	4	ST8576	A	Positive
MC-2-194	Patient57	18/08/13	*K. pneumoniae*	Rectal swab	Surveillance	KPC-2	-	1	ST15	A	Positive
MC-2-198	Patient60	18/08/14	*K. pneumoniae*	Blood	Diagnosis	KPC-2	-	1	ST15	A	Positive
MC-2-200	Patient61	18/08/15	*K. pneumoniae*	Urine	Diagnosis	KPC-2	-	1	ST15	A	Positive
MC-2-203	Patient62	18/08/16	*K. pneumoniae*	Rectal swab	Surveillance	KPC-2	-	4	ST4684	A	Positive
MC-2-206	Patient63	18/08/16	*K. pneumoniae*	Rectal swab	Surveillance	KPC-2	-	1	ST15	A	Positive
MC-2-207	Patient63	18/08/16	*E. coli*	Rectal swab	Surveillance	KPC-2	-	5	ST1236	A	Positive
MC-2-210	Patient64	18/08/17	*K. pneumoniae*	Urine	Diagnosis	KPC-2	-	1	ST15	A	Positive

Strain	Patient	Date	Species	SampleSource	SampleType	*bla*KPC	*bla*CTX.M	PT	ST	Plasmid	NTEKPC
MC-2-209	Patient65	18/08/17	*K. pneumoniae*	Rectal swab	Surveillance	KPC-2	-	1	ST15	A	Positive
MC-2-212	Patient66	18/08/20	*K. pneumoniae*	Rectal swab	Surveillance	KPC-2	-	1	ST15	A	Positive
MC-2-216	Patient67	18/08/21	*K. pneumoniae*	Rectal swab	Surveillance	KPC-2	CTX-M-1	5	ST4685	A	Positive
MC-2-215	Patient69	18/08/21	*K. pneumoniae*	Rectal swab	Surveillance	KPC-2	-	1	ST15	A	Positive
MC-2-222	Patient68	18/08/21	*K. pneumoniae*	Rectal swab	Surveillance	KPC-2	-	1	ST15	A	Positive
MC-2-224	Patient70	18/08/23	*K. pneumoniae*	Rectal swab	Surveillance	KPC-2	-	1	ST15	A	Positive
MC-2-225	Patient71	18/08/23	*K. pneumoniae*	Rectal swab	Surveillance	KPC-2	-	1	ST15	A	Positive
MC-2-226	Patient72	18/08/23	*K. pneumoniae*	Rectal swab	Surveillance	KPC-2	-	1	ST15	A	Positive
MC-2-230	Patient73	18/08/24	*K. pneumoniae*	Rectal swab	Surveillance	KPC-2	-	11	ST307	E	Positive
MC-2-234	Patient74	18/08/27	*K. pneumoniae*	Rectal swab	Surveillance	KPC-2	-	1	ST15	A	Positive
MC-2-237	Patient76	18/08/29	*K. pneumoniae*	Rectal swab	Surveillance	KPC-2	-	1	ST15	A	Positive
MC-2-236	Patient75	18/08/29	*K. pneumoniae*	Rectal swab	Surveillance	KPC-2	-	1	ST15	A	Positive
MC-2-240	Patient75	18/08/29	*E. coli*	Rectal swab	Surveillance	KPC-2	-	3	ST38	F	Positive
MC-2-239	Patient77	18/08/29	*K. pneumoniae*	Rectal swab	Surveillance	KPC-2	-	1	ST15	A	Positive
MC-2-241	Patient78	18/08/30	*K. pneumoniae*	Abcess	Diagnosis	KPC-2	-	1	ST15	A	Positive
MC-2-248	Patient80	18/09/04	*K. pneumoniae*	Rectal swab	Surveillance	KPC-2	-	1	ST15	A	Positive
MC-2-249	Patient81	18/09/04	*K. pneumoniae*	Rectal swab	Surveillance	KPC-2	-	1	ST15	A	Positive
MC-2-250	Patient82	18/09/04	*K. pneumoniae*	Rectal swab	Surveillance	KPC-2	-	1	ST15	A	Positive
MC-2-251	Patient79	18/09/04	*K. pneumoniae*	Urine	Diagnosis	KPC-3	CTX-M-25	9	ST258	C	Negative
MC-2-254	Patient42	18/09/05	*K. pneumoniae*	Rectal swab	Surveillance	KPC-2	-	1	ST15	A	Positive
MC-2-255	Patient83	18/09/05	*K. pneumoniae*	Rectal swab	Surveillance	KPC-2	-	1	ST15	A	Positive
MC-2-258	Patient84	18/09/07	*K. pneumoniae*	Rectal swab	Surveillance	KPC-2	-	1	ST15	A	Positive
MC-2-259	Patient85	18/09/07	*K. pneumoniae*	Sputum	Diagnosis	KPC-2	-	1	ST15	A	Positive
MC-2-257	Patient86	18/09/07	*K. pneumoniae*	Urine	Diagnosis	KPC-2	-	1	ST15	A	Positive
MC-2-263	Patient87	18/09/12	*K. pneumoniae*	Rectal swab	Surveillance	KPC-2	-	1	ST15	A	Positive

Strain	Patient	Date	Species	SampleSource	SampleType	*bla*$_{KPC}$	*bla*$_{CTX.M}$	PT	ST	Plasmid	NTE$_{KPC}$
MC-2-265	Patient88	18/09/13	*K. pneumoniae*	Rectal swab	Surveillance	KPC-2	-	1	ST15	A	Positive
MC-2-271	Patient89	18/09/18	*K. pneumoniae*	Rectal swab	Surveillance	KPC-2	-	1	ST15	A	Positive
MC-2-275	Patient90	18/09/21	*K. pneumoniae*	Rectal swab	Surveillance	KPC-2	-	1	ST15	A	Positive
MC-2-276	Patient91	18/09/21	*K. pneumoniae*	Rectal swab	Surveillance	KPC-2	-	1	ST15	A	Positive
MC-2-278	Patient92	18/09/25	*K. pneumoniae*	Urine	Diagnosis	KPC-2	-	1	ST15	A	Positive
MC-2-282	Patient93	18/09/27	*K. pneumoniae*	Rectal swab	Surveillance	KPC-2	-	1	ST15	A	Positive
MC-2-285	Patient44	18/09/27	*E. coli*	Rectal swab	Surveillance	KPC-2	-	2	ST410	A	Positive
MC-2-291	Patient94	18/10/01	*K. pneumoniae*	Rectal swab	Surveillance	KPC-2	-	1	ST15	A	Positive
MC-2-292	Patient94	18/10/01	*K. pneumoniae*	Rectal swab	Surveillance	KPC-2	-	1	ST15	A	Positive
MC-2-295	Patient95	18/10/04	*K. pneumoniae*	Bronchial aspirate	Diagnosis	KPC-2	-	1	ST15	A	Positive
MC-2-296	Patient96	18/10/08	*E. coli*	Rectal swab	Surveillance	KPC-2	-	1	ST101	A	Positive
MC-2-297	Patient96	18/10/08	*K. pneumoniae*	Rectal swab	Surveillance	KPC-2	-	1	ST15	A	Positive
MC-2-306	Patient48	18/10/09	*K. pneumoniae*	Rectal swab	Surveillance	KPC-2	CTX-M-1	7	ST147	A	Positive
MC-2-304	Patient48	18/10/09	*E. coli*	Rectal swab	Surveillance	KPC-2	-	10	ST1642	H	Positive
MC-2-303	Patient92	18/10/09	*K. pneumoniae*	Rectal swab	Surveillance	KPC-2	CTX-M-9	13	ST37	A	Positive
MC-2-315	Patient96	18/10/20	*E. coli*	Blood	Diagnosis	KPC-2	-	1	ST101	A	Positive
MC-2-316	Patient96	18/10/20	*K. pneumoniae*	Blood	Diagnosis	KPC-2	-	12	ST393	A	Positive
MC-2-328	Patient94	18/10/27	*E. coli*	Rectal swab	Surveillance	KPC-2	CTX-M-1	9	ST1431	A	Positive
MC-2-350	Patient2	18/11/14	*E. coli*	Rectal swab	Surveillance	KPC-2	-	6	ST1193	A	Positive
MC-2-354	Patient97	18/11/26	*K. pneumoniae*	Rectal swab	Surveillance	KPC-2	-	1	ST15	A	Positive
MC-2-360	Patient98	18/12/04	*E. coli*	Rectal swab	Surveillance	KPC-2	-	10	ST1642	H	Positive
MC-2-362	Patient97	18/12/10	*Enterobacter sp.*	Rectal swab	Surveillance	KPC-2	CTX-M-1	6	ND	B	Positive
MC-2-364	Patient55	18/12/12	*K. pneumoniae*	Urine	Diagnosis	KPC-2	-	1	ST15	A	Positive
MC-2-363	Patient99	18/12/14	*K. pneumoniae*	Urine	Diagnosis	KPC-2	-	1	ST15	A	Positive
MC-2-365	Patient57	18/12/16	*K. pneumoniae*	Urine	Diagnosis	KPC-2	-	1	ST15	A	Positive
MC-22-164	Patient100	18/12/19	*K. pneumoniae*	Rectal swab	Surveillance	KPC-2	CTX-M-1	8	ST1564	A	Positive

Strain	Patient	Date	Species	SampleSource	SampleType	***bla*KPC**	***bla*CTX.M**	PT	ST	Plasmid	NTEKPC
MC-2-382	Patient68	19/02/18	*E. coli*	Rectal swab	Surveillance	KPC-2	CTX-M-1	7	ST131	A	Positive
MC-2-387	Patient101	19/02/26	*K. pneumoniae*	Rectal swab	Surveillance	KPC-2, -3	-	10	ST258	D	Positive*

Date: Date of isolation; *bla*KPC: Carriage of corresponding *bla*KPC allele; *bla*CTX-M: Carriage of corresponding *bla*CTX-M allele from CTX-M groups 1, 2, 9 or 25; PT: pulsotype; ST: sequence type; Plasmid: plasmid type according to S1-nuclease digestion; NTEKPC: PCR positive for the presence of a NTEKPC-pMC-2-1 element. *Associated with *bla*KPC-2.
