# Supplementary material for: Clonal Spread and Intra- and Inter-Species Plasmid Dissemination Associated With Klebsiella pneumoniae Carbapenemase-Producing Enterobacterales During a Hospital Outbreak in Barcelona, Spain
Source: Front Microbiol. 2021 Nov 18;12:781127. doi: 10.3389/fmicb.2021.781127 (PMC8637019; doi:10.3389/fmicb.2021.781127)
Supplement: Supplementary file 3 [file Image_1.PDF]

Figure S1

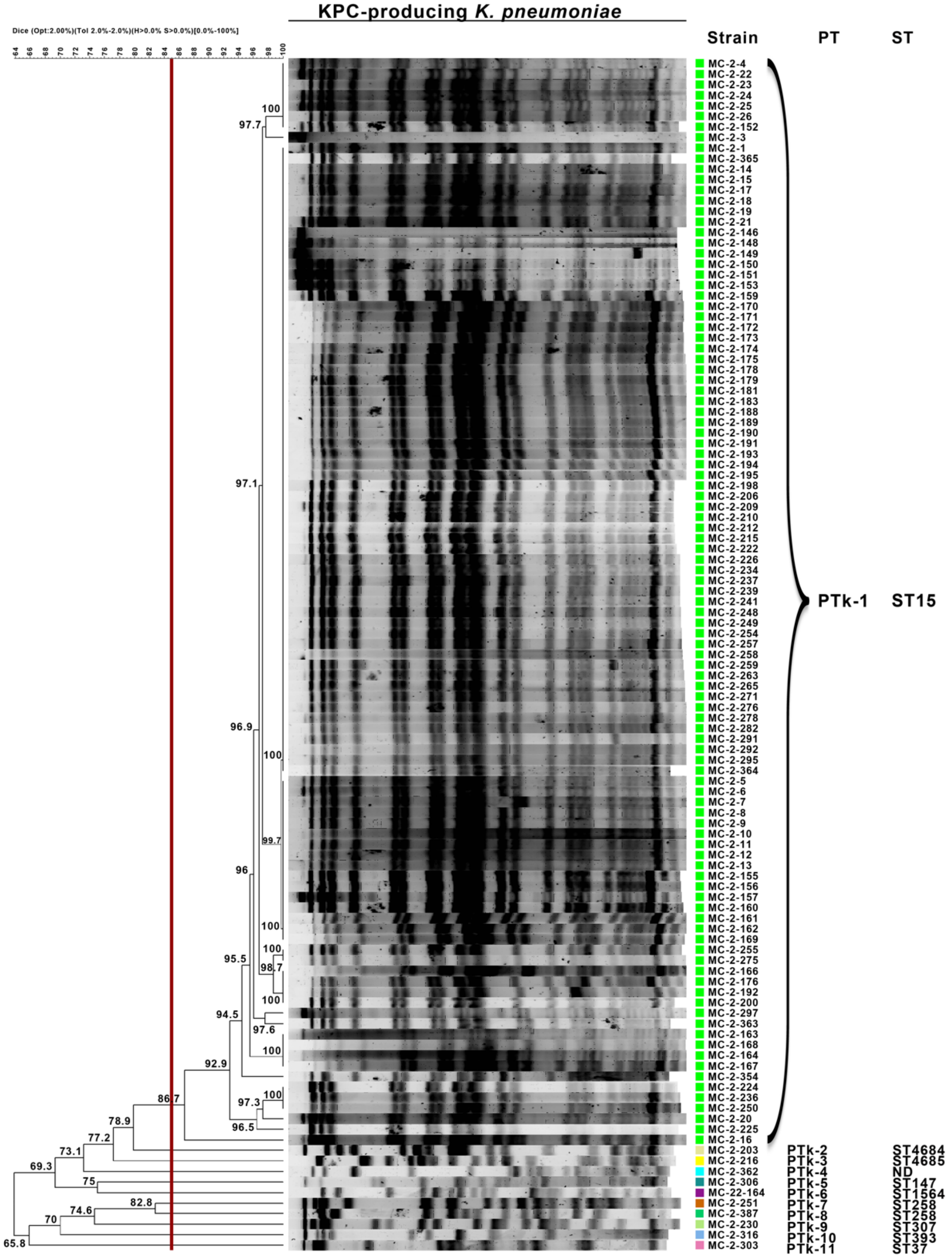

Figure S1. Dendrogram of KPC-producing *K. pneumoniae* and *Enterobacter* sp. isolates recovered in this study from a tertiary hospital in Barcelona. The *K. pneumoniae* pulsotypes (PTk) are shown as well as the corresponding sequence types (ST). The single *Enterobacter* sp. isolate corresponds to PTk-4.
