# Supplementary material for: Clonal Spread and Intra- and Inter-Species Plasmid Dissemination Associated With Klebsiella pneumoniae Carbapenemase-Producing Enterobacterales During a Hospital Outbreak in Barcelona, Spain
Source: Front Microbiol. 2021 Nov 18;12:781127. doi: 10.3389/fmicb.2021.781127 (PMC8637019; doi:10.3389/fmicb.2021.781127)
Supplement: Supplementary file 4 [file Image_2.PDF]

Figure S2

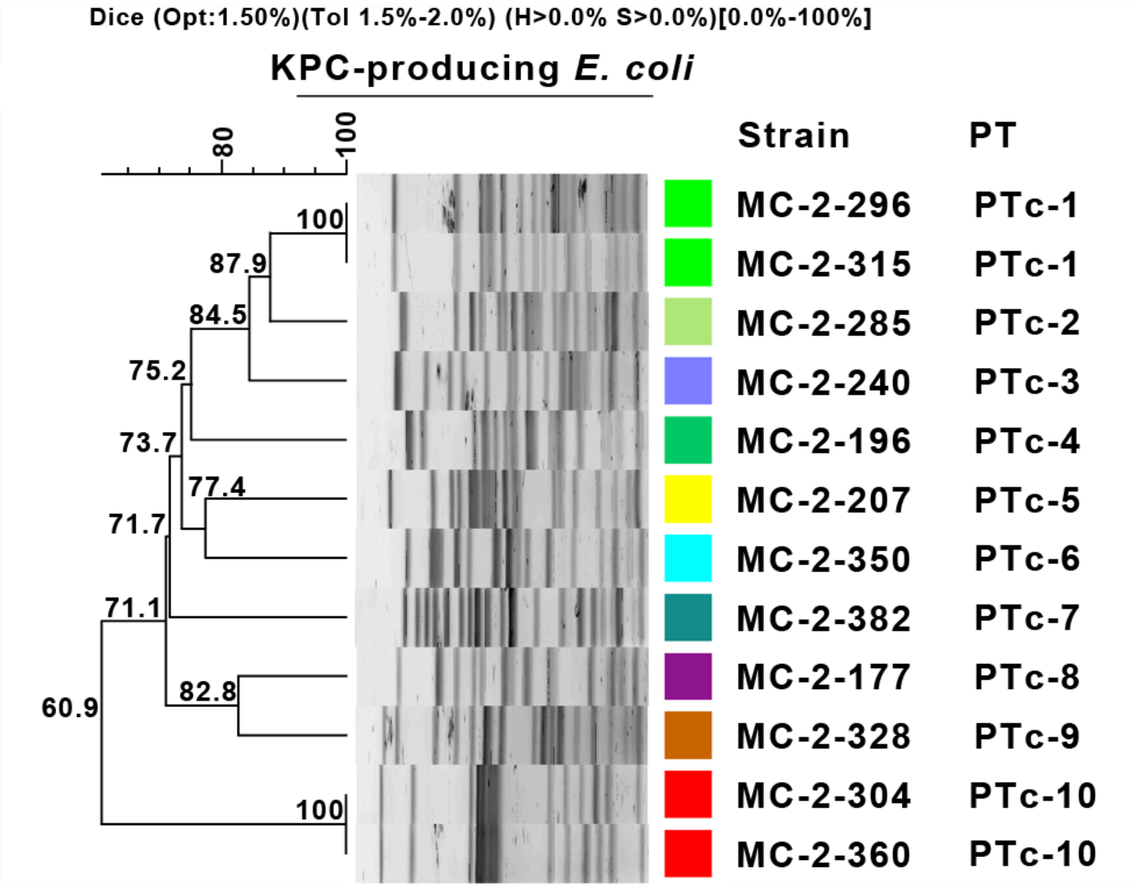

Figure S2. Dendrogram of KPC-producing *E. coli* isolates recovered in this study from a tertiary hospital in Barcelona. The *E. coli* pulsotypes (PTc) are shown.
