# Supplementary material for: Clonal Spread and Intra- and Inter-Species Plasmid Dissemination Associated With Klebsiella pneumoniae Carbapenemase-Producing Enterobacterales During a Hospital Outbreak in Barcelona, Spain
Source: Front Microbiol. 2021 Nov 18;12:781127. doi: 10.3389/fmicb.2021.781127 (PMC8637019; doi:10.3389/fmicb.2021.781127)
Supplement: Supplementary file 5 [file Image_3.PDF]

Figure S3

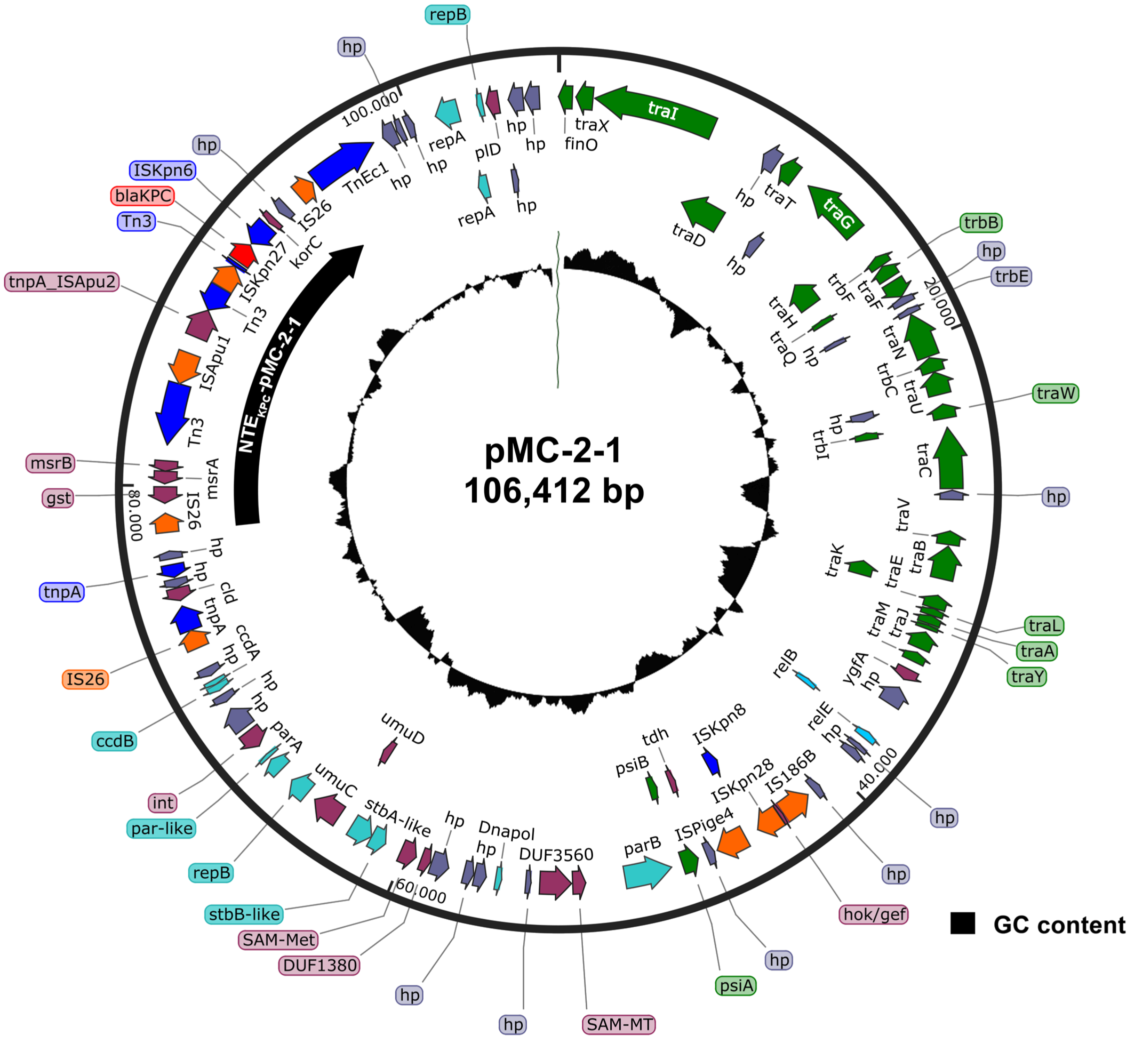

Figure S3. Representation of the IncFIIk plasmid pMC-2-1 (106,412 bp) recovered from the ST15 *K. pneumoniae* MC-2-1 isolate. Arrows are proportional to the lengths of the genes and oriented in the direction of transcription. Red arrows represent resistance genes, orange arrows represent full-length transposon-related genes and ISs, dark blue arrows represent partial or truncated transposon-related genes and ISs, green arrows indicate genes involved in plasmid conjugation and light blue arrows shown genes related to plasmid replication/maintenance. Blue-grey arrows show putative or hypothetical genes and plum arrows show genes involved in other functions. The black arrow highlights the region spanning the NTE$_{KPC}$-pMC-2-1 element flanked by two IS*26*. The inner circle shows the GC content.
