# Supplementary material for: Clonal Spread and Intra- and Inter-Species Plasmid Dissemination Associated With Klebsiella pneumoniae Carbapenemase-Producing Enterobacterales During a Hospital Outbreak in Barcelona, Spain
Source: Front Microbiol. 2021 Nov 18;12:781127. doi: 10.3389/fmicb.2021.781127 (PMC8637019; doi:10.3389/fmicb.2021.781127)
Supplement: Supplementary file 6 [file Image_4.PDF]

Figure S4

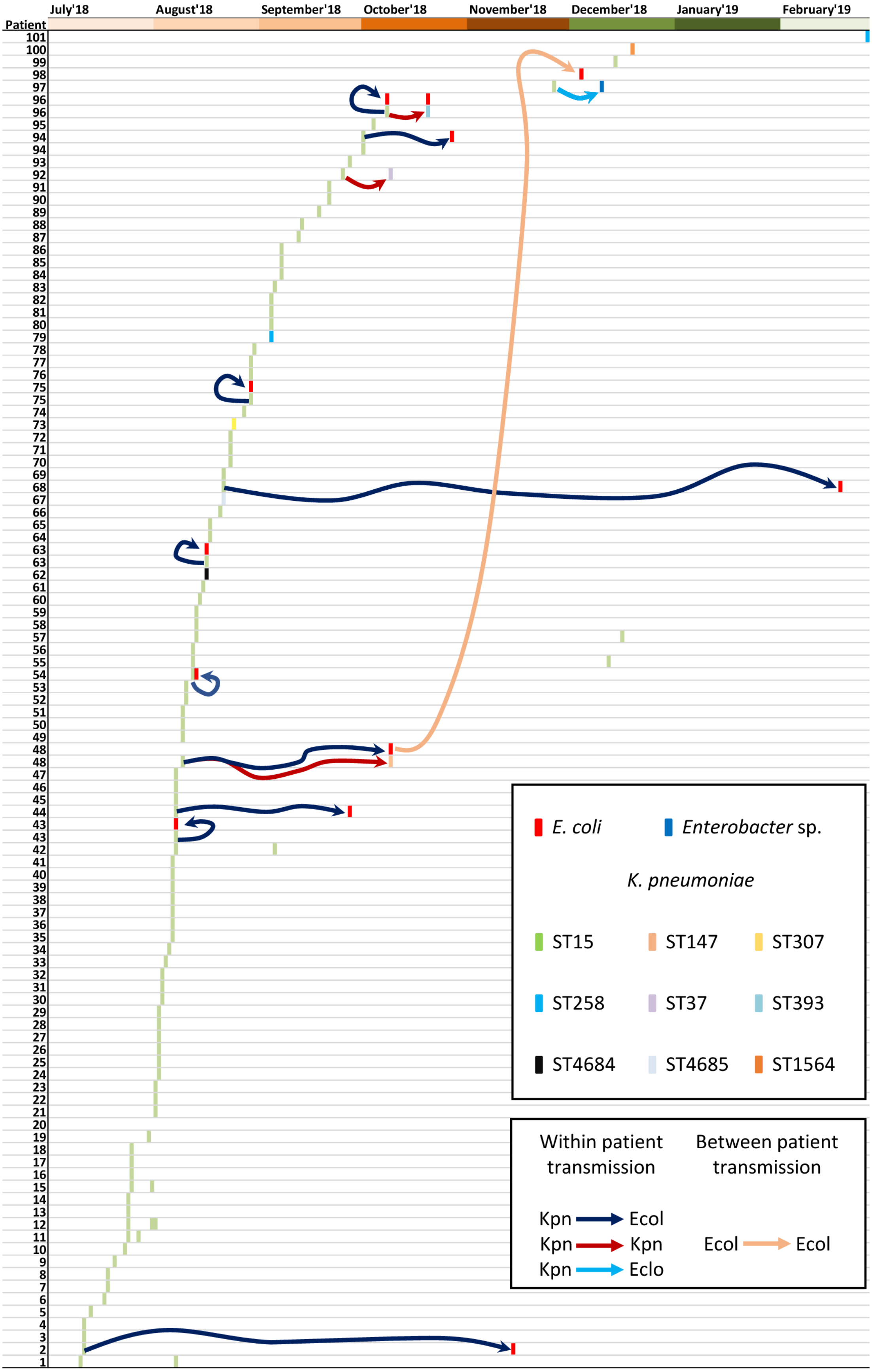

July'18
August'18
September'18
October'18
November'18
December'18
January'19
February'19
Patient
101
100
99
98
97
96
96
95
94
94
93
92
91
90
89
88
87
86
85
84
83
82
81
80
79
78
77
76
75
75
74
73
72
71
70
69
68
67
66
65
64
63
63
62
61
60
59
58
57
56
55
54
53
52
51
50
49
48
48
47
46
45
44
43
43
42
41
40
39
38
37
36
35
34
33
32
31
30
29
28
27
26
25
24
23
22
21
20
19
18
17
16
15
14
13
12
11
10
9
8
7
6
5
4
3
2
1
E. coli
Enterobacter sp.
K. pneumoniae
ST15
ST147
ST307
ST258
ST37
ST393
ST4684
ST4685
ST1564
Within patient transmission
Between patient transmission
Kpn → Ecol
Kpn → Kpn
Kpn → Eclo
Ecol → Ecol

Figure S4. Distribution of patients from July 2018 to February 2019 either infected or colonised by KPC-producing *K. pneumoniae*, *E. coli* or *Enterobacter* sp. isolates. Each row represents a patient and the coloured dots indicate the date of isolation of KPC-producing isolates, coloured according to species. Isolates of *K. pneumoniae* are also colour-coded according to their corresponding sequence type (ST). Putative transmission of plasmid-encoded bla_{KPC} genes within and between patients is outlined by coloured arrows as indicated in the legend. Kpn: *K. pneumoniae*; Ecol: *E. coli*; Eclo: *Enterobacter* sp.
