# Supplementary material for: Clonal Spread and Intra- and Inter-Species Plasmid Dissemination Associated With Klebsiella pneumoniae Carbapenemase-Producing Enterobacterales During a Hospital Outbreak in Barcelona, Spain
Source: Front Microbiol. 2021 Nov 18;12:781127. doi: 10.3389/fmicb.2021.781127 (PMC8637019; doi:10.3389/fmicb.2021.781127)
Supplement: Supplementary file 7 [file Image_5.PDF]

Figure S5

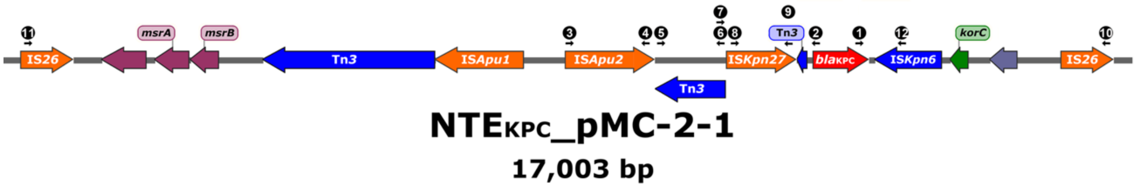

Figure S5. Schematic drawing showing the genetic elements surrounding the bla_{KPC} genes in the NTE_{KPC}-pMC-2-1 variant as well as the annealing sites and orientation for primers listed in Table S1 (Primers 1 to 12).
